# Guidance for ensuring fair and ethical broad consent for future use. A scoping review protocol.

**DOI:** 10.12688/f1000research.51312.1

**Published:** 2021-02-11

**Authors:** Lauren Maxwell, Regina Gilyan, Sayali Arvind Chavan, Laura Merson, Abha Saxena, Rob Terry

**Affiliations:** 1 Heidelberg Institute of Global Health, Heidelberg, 69120, Germany; 2 Max-Weber-Institute of Sociology, Heidelberg, Germany; 3 Institute of Tropical Medicine and Public Health, Berlin, Germany; 4 Infectious Diseases Data Observatory, Centre for Tropical Medicine and Global Health, Oxford, UK; 5 Institut Éthique Humanité Histoire, Geneva, Switzerland; 6 World Health Organization, TDR, the Special Programme for Research and Training in Tropical Diseases, Geneva, Switzerland

**Keywords:** scoping review, data sharing, broad consent for future use, community engagement, benefit sharing

## Abstract

**Introduction: **Broad consent for future use is the reuse of data and/or samples collected by a study by researchers who may not be affiliated with the original study team for purposes that may differ from the objectives of the original study. Sharing participant-level data and samples collected from research participants facilitates reuse and transparency and can accelerate drug or vaccine development, research findings, and translation. Data reuse and synthesis help prevent unnecessary research, thereby respecting research participants time and efforts and building their trust in the research process. Despite these myriad benefits, data and sample sharing represent a significant investment of time for the team that collected the data or samples, and may present additional risks for research participants, including that of re-identifiability and incidental findings, or for the source community. This scoping review will summarize existing guidance on broad consent for future use and highlight evidence gaps related to the ethical, equitable implementation of broad consent for future use.

**Methods and analysis: **We will apply the Arskey and O’Malley scoping review methodology and best practice as outlined in the Joanna Briggs scoping review guidelines.  The research questions have been identified through a literature review and consultation with subject-matter experts. The systematic search will be conducted in three databases using a tailored search strategy. We will search the reference lists of included articles or related systematic reviews for additional citations. The title-abstract and full text screening and charting the data will be conducted independently by two reviewers. Discrepancies will be resolved by a third reviewer. Results will be summarized in narrative form.

**Ethics and dissemination: **This scoping review summarizes findings from existing publications and grey literature rather than primary data and, as such, does not require ethics review. Findings will be disseminated through an open access publication and webinar.

## Background

Research funders ^1^, regulatory agencies, and journals ^2, 3^ are increasingly advocating for and requiring that participant-level data obtained from various forms of health research are shared to maximize their utility. The advantages of sharing data include improving research transparency, facilitating innovation and the conduct of subgroup analyses, and reducing the burden and costs of unnecessary duplication of research ^4, 5^. The public health benefits of sharing data for purposes other than those for which the data were collected, i.e. future use of data, must be balanced with the risk to research participants and the team that collected the data and legal, equity, and ethical concerns, including re-identifiability, incidental findings, benefit sharing, and respecting community values ^6– 8^. These concerns are especially important for researchers and communities in low-and-middle-income countries (LMIC) who may experience a disproportionate proportion of the burden related to data sharing without the same level of benefits as those received by research participants and their communities and researchers in higher income countries ^7, 8^.

Informed consent speaks directly to the core principle of respect for persons and is of central importance to the ethical conduct of human subjects research ^9^. Broad consent for future use is a type of informed consent whereby participants are asked to prospectively agree to the use of data or samples derived from them by unspecified groups for unspecified purposes that are not necessarily related to the objectives of the original study, pending the development or establishment of ethically sound governance for said sharing ^10, 11^. The wording used for broad consent explicitly states or implies that participants will not be re-contacted when sharing their data or samples. While ensuring that the benefits of data or sample sharing outweigh the risks to research participants, researchers and ethics review committees (ERCs) must also take care to ensure that participants understand and agree to data and sample sharing ^12^.

Data sharing can both engender and undermine participants’ trust in the research process. For example, data sharing may accelerate innovations that directly benefit research participants or lead to improved treatments that are only accessible to high income populations ^13^. While the Council for International Organizations of Medical Sciences’ (CIOMS) published guidance for the application of broad consent in 2016, there is a need to supplement this guidance through understanding best practice as it relates to ensuring research participants and their communities understand broad consent for future use and define and realize the benefits of data and sample sharing and that different stakeholders’ interests, risks, and benefits are recognized and addressed in the application of broad consent for future use.

## Study rationale and objectives

In this scoping review, we will summarize empirical findings from primary research and other forms of guidance from international stakeholder groups related to: 1) recommendations around when broad consent should or should not be applied, 2) how to ensure ethical and equitable implementation of broad consent; and 3) and recommended language for broad consent in the health research context. Several prior evidence synthesis efforts have summarized attitudes and best practice related to data sharing. This rapid evidence synthesis will allow for the identification of existing resources for helping ERCs and research teams and communities understand and implement best practice for broad consent for future use and to identify priority areas for future research. This research responds to the challenges faced by researchers and ERCs who must ensure the voluntary and informed participation in the research that they conduct or review and balance the risks and benefits that data sharing represents to different stakeholders while responding to the public health imperative or journal or funder requirements to share data and biological samples derived from clinical trial participants. Findings will be used to develop a research agenda for better addressing key concerns in the ethical and equitable application of broad consent for future use, including re-identifiability, incidental findings, community and research team engagement, and benefit sharing.

## Review methods

Scoping reviews provide a broad overview of existing literature and may be used to identify priority areas for further exploration in a subsequent systematic review ^14^. The key scoping review questions are based on a review of the literature and feedback from subject matter experts and will be updated as needed based on the scoping review findings. This scoping review will follow the Arksey and O’Malley approach to scoping reviews ^15^ and will present the results in keeping with the PRISMA Extension for Scoping Reviews guidance (PRISMA-ScR) ^16^. We will undertake the following steps, from Levac, *et al.*’s recommendations for the conduct of scoping reviews: ^17^

1. clarify and link the purpose and research question (balancing)
2. apply an iterative approach to developing and evaluating the comprehensiveness of the scoping process
3. clarify approach to study selection
4. clarify approach to data extraction
5. qualitative thematic analysis, summarizing results, and clarifying policy, practice, and research implications
6. stakeholder consultation to summarize results and facilitate research translation

This scoping review protocol was developed in keeping with the PRISMA-P guidelines ^18^.

## Stage I: Identifying the research question

The initial review questions were developed with feedback from members of the Reconciliation of Cohort Data in Infectious Disease (ReCoDID) and COVID-19 Clinical Data Research Consortia and the Special Programme for Research and Training in Tropical Diseases (TDR), research groups and an international organization that work on public health data sharing with a focus on ethical and equitable sharing of data and samples collected by research teams in LMIC.

### Review questions

1. What guidelines exist for when investigators should include or not include broad consent for future use in research protocols and informed consent forms?
2. (How) do community members, research participants, research teams, ethics committee members, and regulators understand broad consent and what it means for data, privacy, and the rights of research participants and their communities?
3. When and how should community engagement to ascertain understanding and acceptance of broad consent for future use and meaningful benefit sharing be undertaken?
4. What are the knowledge, attitudes, and practices (KAP) of ethics committees in relation to broad consent?
5. What are the challenges faced by researchers when requesting ethics approval for broad consent for future use of samples and data?
6. How do ERCs evaluate whether investigators have adequately assessed community preferences related to broad consent for future use and benefit sharing?
7. What (ongoing) role should the community/research participants play in the development of governance for sharing different data types (genetic data, identifiable or de-identified clinical-epidemiological data, samples)?
8. What are the recommendations for developing and assessing participants’ understanding of language around broad consent for future use?
9. What options should be provided when presenting broad consent for future use to research participants (tiered, opt in/out)?
10. Should there be a consistent approach to seeking broad consent for future use or does each research project need to determine the best process/language for their research protocol/population? Why/why not?
11. What considerations inform views about whether broad consent is needed for sharing de-identified data/data that are not re-identifiable?
12. How can researchers balance funder and journal requirements for data sharing and institutional requirements around the language for broad consent for future use with community values and preferences for data and sample reuse?
13. What are the competing or conflicting requirements, ethical concerns, risks/benefits, and preferences from different stakeholder groups when considering broad consent for future use? What guidance exists for resolving conflicts or power imbalances in responding to stakeholder priorities?
14. How do researchers navigate the interaction between broad consent for future use, the public health rationale or funder/journal requirements to share data, and research participant and research team member trust?

## Stage II: Identifying relevant studies

In order to identify studies relevant to the subject of this review, we will systematically search the following electronic databases: Ovid (Medline), Cumulative Index to Nursing and Allied Health Literature (CINAHL), and Web of Science. Search strategies include a combination of text and MeSH terms, tailored for each database. Searches will be limited to published papers from the year 2000 onward in order to capture recent broad consent-related studies but will not be restricted by geography or language of publication. We will also search Google and the websites of national or international bodies whose work focuses on data sharing for guidance documents (e.g. Wellcome Trust, Bill and Melinda Gates Foundation, CIOMS). Search results from each database will be imported to EndNote X9 and duplicates will be removed. We will apply snowball sampling wherein we search the reference lists of included studies or related systematic reviews for additional eligible studies. If warranted, the search will be updated and reconducted to further explore emerging research questions. The search strategy for each database is presented in Table 1.

**Table 1.** Tailored search strategy.

	Ovid(Medline)	CINAHL	Web of Science
1	(future use*).ti,ab	TI future use* OR AB ((future use*) N2 (broad OR blanket OR wide) consent) OR AB ((future use*) N2 data shar*)	TI=future use*
2	((broad OR blanket OR wide OR open OR data shar*) adj2 consent).ti,ab.	TI (((broad OR blanket wide OR open OR data shar*) N2 consent)) OR AB (((broad OR blanket OR wide OR open data shar*) N2 consent))	TI=((broad NEAR/2 consent) OR (blanket NEAR/2 consent) OR (wide NEAR/2 consent) OR (open NEAR/2 consent) OR (data shar* NEAR/2 consent))
3	((broad OR blanket OR wide OR open OR consent) adj2 data shar*).ti,ab.	TI (((broad OR blanket OR wide OR open OR consent*) N2 data shar*)) OR AB ((broad OR blanket OR wide OR open OR consent) N2 data shar*))	TI=((broad NEAR/2 data shar*) OR (blanket NEAR/2 data shar*) OR (wide NEAR/2 data shar*) OR (open NEAR/2 data shar*) OR (consent NEAR/2 data shar*))
4	exp animals/ NOT humans.sh.	TI ((animal* OR canine* OR dog* or feline* OR hamster* OR lamb* OR mice OR mouse OR monkey* OR murine OR pig* OR piglet* OR porcine OR primate* OR rabbit* OR rat* OR rodent* OR sheep* OR frog* OR worm* OR trematode) NOT (human* OR patient*))	#1 or #2 or #3
5	1 OR 2 OR 3	#S1 OR #S2 OR #S3	TS=(animal model OR animal* NOT human)
6	5 NOT 4	#S5 NOT S4	#4 NOT #5
7	limit 6 to yr=“2000 - current”	limit #S6 (2000-2021)	Limit #6 (2000-2021)

CINAHL=Cumulative Index to Nursing and Allied Health Literature.

## Stage III: Study selection and eligibility criteria

Citations will be exported to EndNote for deduplication. Title and abstract screening will be conducted independently by two reviewers. Review of full texts for inclusion will be conducted independently by two reviewers and may result in changes to the inclusion criteria, in accordance with the iterative nature of a scoping review. Title-abstract screening, full text screening, and data extraction will be managed in Covidence ^19^. Inter-reviewer disagreements in the title-abstract or full text screening will be resolved by a third reviewer. ****

### Inclusion criteria

Included studies will be published from 2000 onwards and will report on primary research with research participants or stakeholders (e.g. policy makers, researchers, ERC committee members) or guidance documents from international bodies and guidelines development groups, e.g., the WHO, the Pan American Health Organization, CIOMS, Wellcome Trust, the Global Research Collaboration for Infectious Disease Preparedness, Canadian Institutes for Health Research, Bill and Melinda Gates Foundation, and National Institutes of Health. Studies may apply qualitative, quantitative, or mixed methods approaches to understanding broad consent for future use. Quantitative studies may include interventions or observational studies.

### Exclusion criteria

Systematic reviews, commentaries, and editorials or other forms of research that do not present primary research or reflect the opinions of a larger stakeholder group will be excluded. Editorials and commentaries represent the opinions of individual authors rather than an internationally recognized group or guidelines development organization. Primary studies that report on data sharing preferences or practices but that do not specifically address broad consent for future use and guidance or research from fields that are not related to public health will be excluded. Lastly, manuscripts and guidance published prior to 2000 be excluded.

## Stage IV: charting the data

We will develop and pilot a charting form to facilitate the descriptive synthesis of scoping review findings prior to beginning the charting process. We will extract the key data detailed in Table 2 from included studies to facilitate the summary of available guidance and existing gaps in the literature. We will pilot the data extraction forms using two-three full text articles. Data extraction and charting will completed independently by two reviewers, in keeping with best practice. We will not include an assessment of the quality of included studies. The full text of included studies will be exported to MAXQDA 2020 ^20^ for thematic analysis using deductive codes and inductive codes that emerge from the review of full text articles.

**Table 2.** Proposed data charting fields.

Study	• author • publication year • study type and approach (observational, intervention. qualitative, quantitative, mixed methods, guidance document that is not grounded in empirical research)
Population	• location • year of study • participant enrollment criteria • source population • actual or theoretical participants in broad consent-related initiative
Broad consent	• Definition of broad consent for future use • data types examined (e.g., genetic, samples, medical records) • approach to broad consent (e.g., tiered, dynamic consent) • rationale for inclusion/exclusion of broad consent in consent forms
Findings	• findings, including key findings related to acceptability of broad consent for future use or acceptability/feasibility of different consent models • recommendations related to developing and assessing participants’ understanding of broad consent language • knowledge attitudes and practices of researchers, funders, ethics committee members, research teams, research participants, as related to broad consent for future use
Challenges, evidence gaps	• Challenges confronted by researchers, ERC members, research participants, in understanding and evaluating broad consent for future use • Gaps in the evidence base for current recommendations

## Stage V: collating, summarising and reporting results

Results from the data charting work will be presented in narrative form. A PRISMA flow diagram will be used to report the final number of included studies in the review and basic study information will be presented in table format. The results summary will present both quantitative and qualitative aspects of included studies.

## Stage VI: consultation, patient & public involvement

A group of experts in the field of data sharing and broad consent for future use will inform the analysis and summary of findings and assist with the dissemination of any recommendations. Research findings will be shared publicly through a webinar and through the websites of partner institutions.

## Ethics and dissemination

No ethical approval is required for this systematic review as published articles, rather than primary data, will be used in the analyses. Findings will be presented in an Open Access publication and disseminated on the COVID-19 Clinical Data Consortium’s website and through Twitter.

## Study status

The current study status and anticipated timeline are presented in Table 3.

**Table 3.** Study status at time of protocol submission and anticipated timeline.

Preliminary search	Completed
Pilot of search strategy	Completed
Implementation of search	February 2021
Title-abstract screening	February 2021
Full text screening	February 2021
Development and piloting of data charting tool	February 2021
Charting the data	March 2021
Publication of results	April 2021

## Conclusion

The global coronavirus disease 2019 (COVID-19) pandemic has foregrounded the public health importance of sharing participant-level data and samples. In response to the urgent need for supplementary guidance to support the inclusion of broad consent for future use in research protocols and informed consent forms, we will review and summarize empirical research and guidance related to broad consent for future use of data or samples collected in health-related research. By applying best practice to a rapid scoping review, we hope to facilitate the ethical and equitable application of broad consent for future use in the public health research response to COVID-19.

## Data availability

### Underlying data

No data are associated with this article.
