# Supplementary material for: Guidance for ensuring fair and ethical broad consent for future use. A scoping review protocol
Source: F1000Res. 2021 Feb 11;10:102. [Version 1] doi: 10.12688/f1000research.51312.1 (PMC8063550; doi:10.12688/f1000research.51312.1)
Supplement: Peer review history

# Peer review: Reviewer response for version 1

**DOI:** 10.5256/f1000research.54466.r81730

**Published:** 2021-04-16

**Authors:** Limbanazo Matandika

**Affiliations:** 1 Centre for Bioethics in Eastern and Southern Africa, College of Medicine, University of Malawi, Blantyre, Malawi

Overall this scoping review that’s aims at summarizing existing guidance on broad consent for future use and highlight evidence gaps related to the ethical, equitable implementation of broad consent for future use is reasonably designed and a potentially important contribution to the literature. That said, there is a shortcoming in one of the strategies for data collection stage VI of the protocol there is no clarification whether this is part of the methods or a procedure. The author needs to clearly state if the consultation, patient and public involvement aspect is aimed at collecting data as part of confirming the findings or not? There is need for a more comprehensive outline of how this will be achieved.

 Revisit the definition of Broad consent in the first sentence of the abstract. It seems there is a mix-up on what broad consent is and its benefits.

 The strongest points in this article is the use of the PRISMA-P guidelines that has outlined 5 stages for data collection in the scooping exercise. The methods do not only outline literature review aspects but incorporate engagement with relevant stakeholders in the practice. Involvement of stakeholders as part of data collection will allow the validation of the findings from literature and outline the grey areas in the use of Broad Consent in research.

 The paper though could have been strengthened much better if the data collection method included exploring of perspectives from sample donor’s themselves especially in times of a global pandemic like COVID-19. Literature on sample use may provide insights on regional epidemic/pandemics. COVID-19 is a global pandemic hence it's very critical to understand how perspectives on use of sample could differ. Therefore, the researcher may need to consider how insights from a wider perspective would be relevant to the global pandemic.

Is the study design appropriate for the research question?

Yes

Is the rationale for, and objectives of, the study clearly described?

Yes

Are sufficient details of the methods provided to allow replication by others?

Yes

Are the datasets clearly presented in a useable and accessible format?

Yes

Reviewer Expertise:

My expertise is in research ethics. Areas include; biobanking, informed consent (communication strategies and various models of Informed consent).

I confirm that I have read this submission and believe that I have an appropriate level of expertise to confirm that it is of an acceptable scientific standard.

# Peer review: Reviewer response for version 1

**DOI:** 10.5256/f1000research.54466.r79603

**Published:** 2021-03-01

**Authors:** David Osrin

**Affiliations:** 1 Institute for Global Health, University College London, London, UK

This protocol for a scoping review is concise, clear, and timely. The authors will follow recommended guidelines for scoping reviews.

 General comments

1. One thing that strikes me — and will certainly have been discussed by the authors— is the inclusivity of the search terms. Adding search terms sequentially reduced the returned list to as few as 2 or 0 publications, whereas keeping the search broad returned manageable numbers under 100.

Specific comments:

 Abstract

1. The first sentence doesn’t quite work: “Broad consent for future use is the reuse of data and/or samples collected by a study by researchers who may not be affiliated with the original study team for purposes that may differ from the objectives of the original study.” The sentence is saying what is meant by future use, not broad consent for it.
2. “…participants time and efforts…” Apostrophe “participants’”

Background

1. P3 column 2 line 1: there is an extra “and”
2. P5 Stage 4: “Data extraction and charting will completed:” missing “be”
3. P5 Stage 4: “We will not include an assessment of the quality of included studies.” I’d suggest a sentence saying why not.

Is the study design appropriate for the research question?

Yes

Is the rationale for, and objectives of, the study clearly described?

Yes

Are sufficient details of the methods provided to allow replication by others?

Yes

Are the datasets clearly presented in a useable and accessible format?

Not applicable

Reviewer Expertise:

Public health, ethics of data sharing

I confirm that I have read this submission and believe that I have an appropriate level of expertise to confirm that it is of an acceptable scientific standard.
